# Analysis of Impact Characteristics and Detection of Internal Defects for Unidirectional Carbon Composites with Respect to Fiber Orientation

**DOI:** 10.3390/polym13020203

**Published:** 2021-01-08

**Authors:** Sun-ho Go, Alexandre Tugirumubano, Hong-gun Kim

**Affiliations:** 1 Mechanical Testing Center, ITEL Co. LTD., Cheong-ju 28220, Korea; sh.go@itel.re.kr; 2 Institute of Carbon Technology, Jeonju University, Jeonju-si 55069, Korea; alexat2020@jj.ac.kr; 3 Department of Mechanical and Automotive Engineering, Jeonju University, Jeonju-si 55069, Korea

**Keywords:** drop-weight impact, unidirectional carbon composites, orientation angle, internal defect, impact

## Abstract

With the increasing use of carbon fiber reinforced plastics in various fields, carbon fiber composites based on prepregs have attracted attention in industries and academia research. However, prepreg manufacturing processes are costly, and the strength of structures varies depending on the orientation and defects (pores and delamination). For the non-contact evaluation of internal defects, the lock-in infrared thermography was proposed to investigate the defects in the composites subjected to the compression after impact test (CAI). The drop-weight impact test was conducted to study the impact behavior of the composites according to fibers orientation for composite fabricated using unidirectional (UD) carbon fiber prepregs. Using CAI tests, the residual compressive strengths were determined, and the damage modes were detected using a thermal camera. The results of the drop weight impact tests showed that the specimen laminated at 0° suffered the largest damage because of susceptibility of the resin to impact. The specimens with 0°/90° and +45°/−45° fibers orientation exhibited more than 90% of the impact energy absorption and good impact resistance. Furthermore, the specimens that underwent the impact tests were subjected to compressive test simultaneously with the lock-in thermography defects detection. The results showed that internal delamination, fibers splitting, and broken fibers occurred. The temperature differences in the residual compression tests were not significant.

## 1. Introduction

In recent years, the research on composites materials and the industrial applications has rapidly expanded with the aim of decreasing the energy consumption and the development of new materials with lightweight characteristic. Among them, carbon fiber-reinforced plastics (CFRPs) have been used in several industries that require weight reduction, due to their higher specific strength, higher specific stiffness, low density, their excellent chemical resistance, and electrical properties when compared with existing metallic materials [1,2,3]. The CFRPs are the composites where the carbon fibers are used as strengthening elements to provide high strength in polymer matrix. The long carbon fibers are the most used to fabricate the composites for structural applications. The common method of fabricating these composites is through laminating the dry carbon fibers fabrics and applying the polymer as binding element or laying up the carbon fibers fabrics pre-impregnated with resin (prepreg). The carbon fiber fabrics may be either unidirectional (UD) or woven. Unlike the woven carbon fiber fabrics that have large gaps between the fibers due the interlacing of the yarns (warp and wefts), in the unidirectional carbon fiber fabrics, the gaps are almost absent because the fibers are tightly assembled in single and parallel direction [4]. The properties of the CFRP depend on the lamination sequence, orientation of the fibers, and manufacturing methods.

Due to the development of three-dimensional automatic fiber placement technologies, carbon composites with prepregs have attracted significant research attention. However, the processes that involve prepregs are costly, and the strength of carbon fiber composites varies significantly depending on the fiber orientation and defects, such as delamination and pores. Those defects can degrade the quality and the performance of the composites. The production of good quality composites involves the experience, suitable manufacturing process, and quality testing. Hence, a non-destructive evaluation method is urgently needed to investigate the quality of the produced composites [5,6].

Among the existing non-destructive evaluation methods for CFRPs, defect imaging based on ultrasonic C-scans has been effectively employed [2]. Given that the method is a contact type, there are size and process limitations. The infrared (IR) thermography using IR thermal imaging camera is the technique that allows the determination of the surface temperature distribution of an object based on IR waves emitted from the surface. This technique has been extensively implemented in non-contact temperature measuring equipment [7]. Infrared thermography involves the detection of radiant energy from the surface of an object, and the conversion of that energy into the temperature domain, thus revealing the thermal distribution in a real-time image. However, defect detection for objects with high thermal diffusion coefficients is challenging and can be realized by the application of the lock-in method [3,7].

The detection mechanism of the lock-in system is based on the synchronization of the heat source signal with the noise from an IR thermal imaging camera that measures the IR radiation generated from the surface of a specimen under periodic loading. The differences between the heat source signal and response signal of the object are then obtained and analyzed.

In particular, given that CFRPs are susceptible to impacts, research on the improvement of their impact performance is required. It is common knowledge that the damage due to the impact of composite materials varies with respect to the impact velocity, and that local impact damage can be attributed to the insufficient impact response time of composite materials at low impact speeds of 1–100 m/s, and high impact speeds [8,9,10,11,12]. In the case of low-speed impacts, non-visible microscopic damage mainly occurs due to impact. In addition, given the high elasticity of CFRPs, the kinetic energy of the impactor is stored in the CFRP as absorbed energy under low-speed impacts, and it is then re-transferred to the kinetic energy in the impactor through elastic restoration [8,13,14,15,16,17,18,19,20,21,22].

This work aims to study the mechanical impact performance of the CFRP through the drop-weight impact testing according to the orientation of unidirectional carbon fiber fabrics. Further, the non-destructive inspections of the defects caused by impact test were performed using lock-in infrared thermography method. The compression after impact test was also conducted to evaluate the residual compressive strength of the composites. The infrared camera was used to assess the temperature variation at the failure of the composites during the testing.

## 2. Materials and Method

### 2.1. Material and Composite Manufacturing

**Figure 1:**
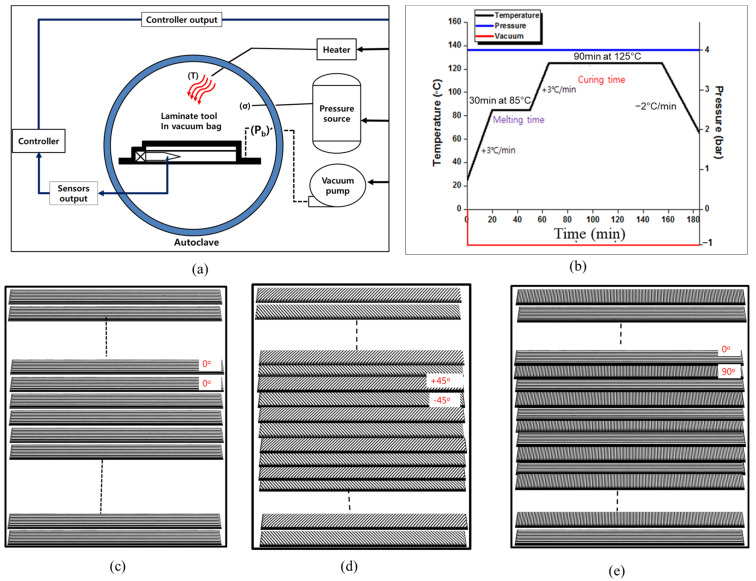
Molding process; (**a**) Flow diagram of autoclave molding method; (**b**) Cycle of molding and curing conditions, (**c**–**e**) Stacking sequence diagram of [0°]_36_, [0°/90°]_18_, [+45°/−45°]_18_, respectively.

The material used in this study was UIN200C, which is a unidirectional carbon fiber prepreg purchased from SK Chemical, Seongnam-si, Korea. The laminated prepreg was manufactured using the autoclave molding process, as shown in Figure 1a,b presents the cycle of molding and curing conditions, and Table 1 presents the physical properties of the material. The following three configuration of composites with different orientation of the fibers were fabricated (Figure 1c,d): a composite with 36 plies unidirectional prepreg at [0°]_36_, a composite with 34-plies prepreg cross-laminated at [0°/90°]_18_, and a composite with 36 plies prepregs cross-laminated at [+45°/−45°]_18_. For each lamination configuration, the composite plate with dimensions of 450 mm × 420 mm were fabricated. For the material properties testing, the test specimens were cut from the fabricated composite plate. Four specimens for each composite were tested to evaluate the impact and compression after impact behavior of the composites.

### 2.2. Drop-Weight Impact Test

The drop-weight impact test was conducted for the fabricated composites. In previous studies, the drop-weight impact failure trend of polymer materials has been found to occur through either the matrix cracking damage of the single layer, which is internal damage; or delamination from the interface; or the external damage. Thus, there exist three cases of failure mode in composite due to drop-weight impact [13,21,23]. Those cases are puncture failure mode, crack failure mode, and brittle fracture mode. In the case of the puncture failure mode (P-mode), the full-penetration failure geometry can be observed, in which the diameter of failure site is almost equal to that of the weight. This failure behavior can be observed in polycarbonate and polyethylene. In the case of crack failure mode (C-mode), a failure is generated at the deformation zone around the weight collision site and cracks propagate toward the surrounding areas. The C-mode can be observed in polypropylene and propylene-ethylene block co-polymers. In the case of the brittle fracture mode (B-mode), failure occurs with radial crack propagation from the center of the specimen. Moreover, this failure behavior is observed in polystyrene, and the brittle failure mode has slight influence on the absorption energy [13,15,16,17,23].

In this study, for the drop-weight impact test, the test conditions and specimen specifications were selected in accordance with the ASTM D 7136 standard. The impactor was hemispherical with diameter of 16 mm. The impact test specimen with 100 mm × 150 mm was clamped in the impact support fixture that had a window size of 75 mm × 125 mm. 

**Figure 2:**
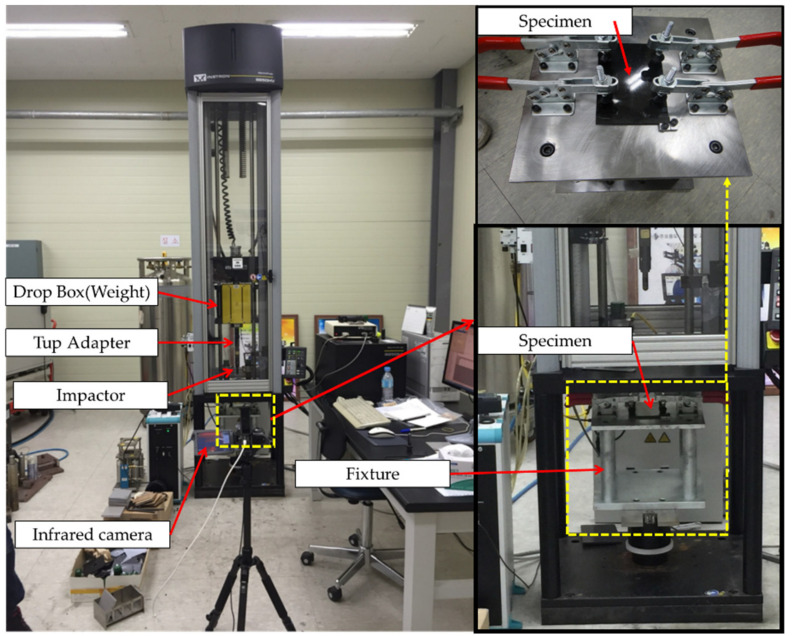
Experimental setup.

The analysis was conducted for three specimens per type of composite. The impact absorption efficiency,$\;e_{abs}$, of each material was analyzed using the following Equation (1):

(1) $$e_{abs}\left(\%\right)=\frac{J_{a}}{J_{i}}\times 100$$

where $J_{i}$ and $J_{a}$ are the impact energy (in Joule) and the impact absorption energy (in Joule), respectively. Given that the impactor weight, speed, and drop height are critical parameters in the drop-weight impact test, these parameters were calculated and applied using the formulas specified in the ASTM D 7136 standard. The weight used was 4.23 kg, and the test was conducted with drop height of 0.92 m and drop speed of 4.21 m/s. Figure 2 presents the test equipment and components used. The IR thermal image camera FLIR A655sc (FLIR Systems Inc. Willsonville, OR, USA), with a temperature measurement range of −40 °C to 150 °C and 100 °C to +650 °C and accuracy of ±2 °C or ±2% of reading, was used to convert the radiant energy generated during impact into the temperature domain, and to analyze the relationship between the impact energy and heat energy. In addition, the mode that leads to failure was determined based on the thermal distribution of the impact energy. The temperature results were compared and analyzed by determining the difference between the room temperature before the test and the maximum temperature during breakage (ΔT). 

### 2.3. Compression-after-Impact Test

**Figure 3:**
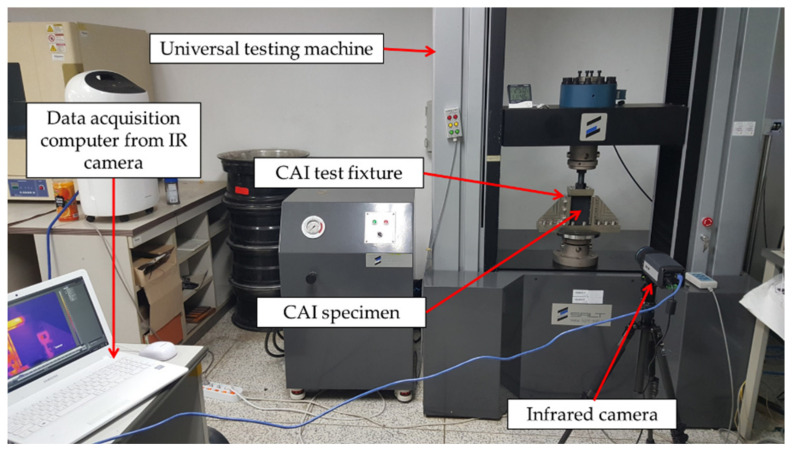
Compression after impact (CAI) experimental setup.

The compression test was conducted on the specimen that underwent into impact test is mainly referred to the compression-after-impact (CAI) test. The CAI was conducted in accordance with the ASTM D 7137 standard. The size of the CAI test specimens was the same as the drop-weight specimens (100 mm × 150 mm). It is common knowledge that the CAI test is conducted for examining the matrix cracking and fiber breakage after impact damage with respect to the lateral shear and vertical stress of laminated composites, in addition to the compressive strength after impact [24,25,26,27]. In particular, delamination is known to reduce the compressive strength of laminated composites by 40–60%. The CAI strength was calculated based on the compressive load and cross-sectional area of the specimen, using the installed jig, as shown in Figure 3. During the test, the IR thermal image camera was installed and used to identify the temperature distribution with respect to breakage. The temperature results were then compared and analyzed based on the difference between the room temperature and the maximum temperature during breakage (Δ*T*). In addition, the strength degradation after impact was compared with the compressive strength of WSN-3K, which was approximately 440 MPa. 

### 2.4. Lock-In Thermography

**Figure 4:**
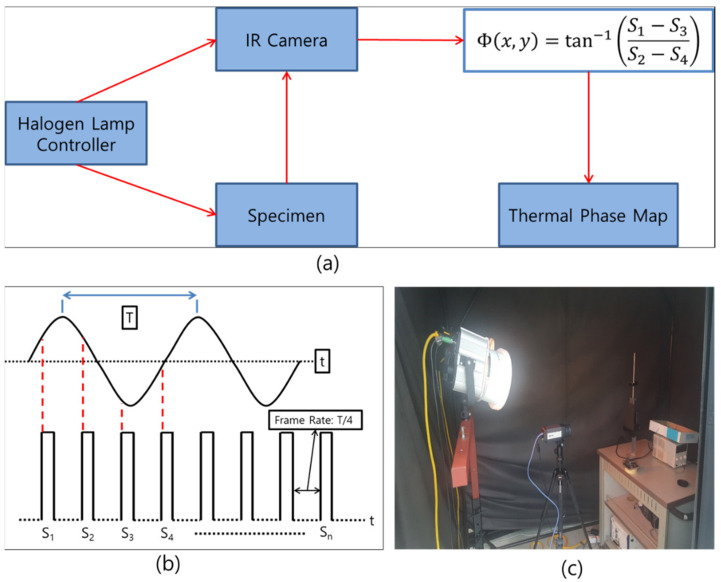
Lock-in method: (**a**) flowchart; (**b**) diagram and (**c**) experimental Setup.

The lock-in method was considered in this study for non-destructive inspection (Figure 4), which is referred to as lock-in IR thermography. This method was employed to obtain the response signal of the object by synchronizing a halogen lamp (heat source) with the IR detection element of the thermal imaging camera, and to realize the detection based on the changes in the infiltrated heat source signal. In this case, the one-dimensional heat conduction Equation (2) in the solid was used as follows [7,14]:

(2) $$\frac{\partial T}{\partial t}=\frac{k}{\rho C_{p}}\;\frac{\partial^{2}T}{\partial x^{2}}$$

In Equation (1), *T* is the temperature, *t* is the time, *k* is the thermal conductivity, ρ is the density, $C_{p}$ is the specific heat, and *x* is the distance in the heat flow direction. The solution of Equation (2) based on the harmonic function can be expressed as Equation (3) [7]:

(3) $$T\left(x,t\right)=\;T_{o}e^{-x/\mu }cos\left(\omega t-\frac{x}{\mu }\right)$$

where *µ* is the thermal diffusivity and *T_o_* is the initial temperature, and ω is the modulation frequency.

The lock-in method can improve the detection sensitivity by extracting the phase from the measurement results using Equation (3) and minimize the defect detection error due to the non-uniformity of the surface emissivity. The phase in Equation (3) can be used to obtain IR detection signals *S*_1_*, S*_2_*, S*_3_*,* and *S*_4_, which are consecutive at the intervals of the λ/4 period of the heat source, by synchronizing the external heat source with the IR detection element. Finally, the results can then be obtained using Equations (4) and (5) [7]:

(4) $$\begin{array}{c} S_{1}=\;T_{o}e^{-x/\mu }cos\left(\omega t-\frac{x}{\mu }\right)\\ S_{2}=\;T_{o}e^{-x/\mu }cos\left(\omega t-\frac{x}{\mu }-\frac{\pi }{3}\right)\\ S_{3}=\;T_{o}e^{-x/\mu }cos\left(\omega t-\frac{x}{\mu }-\pi \right)\\ S_{4}=\;T_{o}e^{-x/\mu }cos\left(\omega t-\frac{x}{\mu }-\frac{3\pi }{2}\right) \end{array}$$

(5) $$\emptyset =\frac{x}{\mu }=\tan^{-1}\left(\frac{S_{4}-S_{2}}{S_{1}-S_{3}}\right)$$

## 3. Results and Discussion

### 3.1. Results of Impact Test

**Figure 5:**
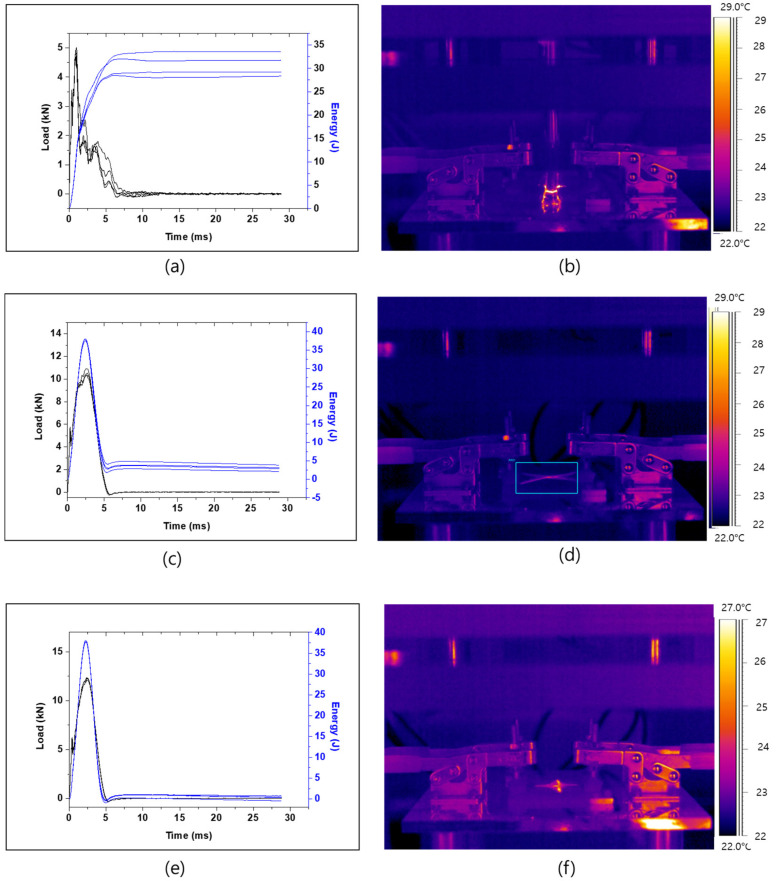
Impact test results for energy and load of specimens with respect to time (left), and thermal distribution image (right): (**a**,**b**) specimen laminated at an angle of 0°; (**c**,**d**) specimen laminated at an angle of +45°/−45° and (**e**,**f**) specimen laminated at an angle of 0°/90°.

The following results (Figure 5a,b and Table 2) were obtained for the composite in which the unidirectional prepregs were laminated at an angle of 0°. Given that the carbon fibers were laminated in one direction, the specimen underwent breakage in the direction of the carbon fiber after impact. The same conclusion could be obtained from the thermal distribution image. The impact absorption efficiency was 17.96%, which indicates that 17.96% of the applied impact was absorbed by the specimen whereas the other energy was transformed in heat energy as temperature variation found to be high. However, the sample was completely fractured after being impacted. Practically, in the case of the carbon composite with lamination in 0° direction, the epoxy support was in the transverse direction, and not in longitudinal direction (the fiber direction). Moreover, due to the brittleness of the resin, breakage of the matrix easily occurred.

Figure 5c,d and Table 3 present the test results for the composite in which carbon fibers were cross-laminated at an angle of +45°/−45°. A different trend from that of the composite laminated in one direction was observed (Figure 5c). The impact absorption efficiency was 92.11% on average, which indicates that the applied impact energy by the impactor to the composites were attenuated up to 92.11% by the composites. The thermal image (Figure 5d) shows the cracks generated during the transmission of the impact load from the impact site across the composites. The results showed that the cracks tended to propagate in a similar direction to the fiber orientation. This means that the damages were mainly due to breakage of brittle epoxy matrix which caused the progressive debonding (separation) of the adjacent fibers close to the impact site in the fiber orientation.

Figure 5d–f and Table 4 present the test results of the composite in which carbon fibers were cross-laminated in the 0°/90° orientation. As for the composites with +45°/−45° orientation, Figure 5d showed different trend from that of the composite laminated in one direction was observed (Figure 5a). The average impact absorption efficiency was 98.72%, which indicates that the applied impact was blocked by the composite or re-transferred to the impactor. The thermal distribution image reveals the generation of cracks during the transmission of the impact loading from the impact site through the thickness of the composites. Different from the other configurations, it could be seen that the propagation of cracks due to the impact test were in two orthogonal directions with long and extended crack propagation in direction perpendicular to the fiber’s orientation. However, from the thermal image (Figure 5d), it can be seen that the very critical damages occurred in the vertical direction (fiber orientation) which can be attributed to breakage of both matrix and fibers. Figure 5b,d,f showed that at the impact site, the damage was severe. That can be associated to the fracture of fiber and matrix at that location on the side of the impactor, and the other damages such as the push-out delamination [5] due to the transmission of impact load through the composites around the impact location.

### 3.2. The Thermography Results

**Figure 6:**
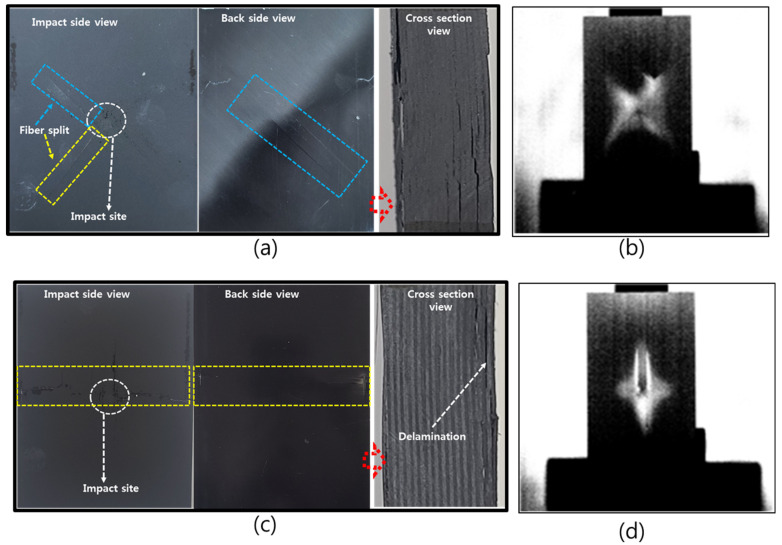
Digital camera images for external view (left) and lock-in thermography image for defects detections (right): (**a**,**b**) Specimen lamination in +45°/−45° and (**c**,**d**) Specimen lamination in 0°/90°.

Figure 6 presents the images of the analyzed specimens after the drop-weight impact test using the lock-in method for the specimens +45°/−45° and 0°/90°. In the case of the specimen laminated with all fibers laminated in 0°, the non-destructive inspection method was not conducted because the composite with all plies oriented in 0° was completely broken by the drop-weight impact test. The lock-in non-destructive test was performed to conform the internal damage of the test specimen after the actual drop-impact test. From the images shown in Figure 6a,c, the damage could be observed at the impact side of back side of the composites. The cross-section of the specimens at the center of impact location showed that the delamination and fiber breakage occurred in all composites. It can be seen that the delamination mostly occurred from the back side which can be attributed to impact load transfer as form of tensile stress wave to the back face of the composites. The critical damages were observed at the interlayers and through the fibers splitting. 

The images on the left-hand side, Figure 6b,f, are the images of the specimens after impact, whereas those on the right-hand side are the images of the same specimens analyzed using the non-destructive inspection method. After the impact test, the mark left by the impactor and small cracks could be observed with the naked eye to assess the degree of the external damage (Figure 6a,c). However, the internal damages could be observed by using the lock-in non-destructive test. It can be seen that the lock-in non-destructive technique, the extension of damage, and the overall geometry of the damage in the composite could be detected. The geometry of damage depended on the stacking structure of the composites and in both, the long axes (or the diagonals) of the damage shapes were oriented in the same direction as the principal direction of laminated plies. This indicated that the damage propagation in the CFRP composites (with long fibers) mostly follows the fiber orientation. This behavior was believed to be associated with the progressive separation of carbon fibers along their directions due to the breakage of brittle epoxy matrix and the crack propagations from the impact location. 

### 3.3. Results of CAI Test

**Figure 7:**
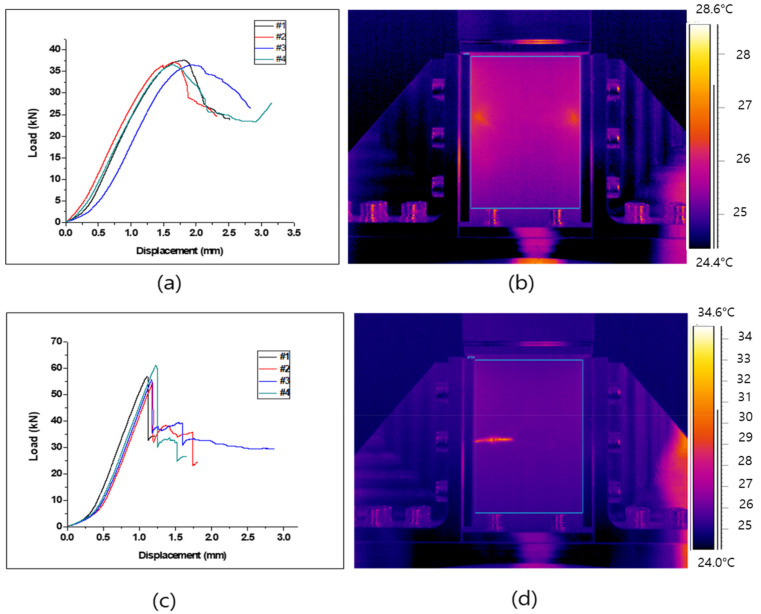
Load-displacement curve of the cai test (left) and thermal image of cai test (right): (**a**,**b**) specimen lamination in +45°/−45° and (**c**,**d**) specimen lamination in 0°/90°.

The CAI test results shown in Figure 7a,b and Table 5 of the specimens laminated at angles of 0°/90° and +45°/−45°. The CAI test was not conducted on the specimen laminated at an angle of 0°, as it was completely destroyed by the drop-weight impact test. The load-displacement curves (Figure 7a,c) showed a non-linearity behavior at the beginning of the CAI test. This was evidently due to the presence of irregular cracks in the composites which were created by the impact test. Then, at the beginning of the CAI test, those cracks were gradually closed due to the applied load. After the cracks were closed, a drastic increase of the load with little displacement could be observed.

In the case of the specimen laminated in +45°/−45° directions, the residual compressive strength was 67.02 MPa, and the temperature difference was 2.56 K, thus indicating a negligible temperature difference. This can be attributed to the lack of supporting fibers in the load direction, and the orientation of fibers at angles of ±45°.

In the case of the specimen laminated 0°/90° in directions (Figure 7c,d and Table 5), the residual compressive strength was 113.78 MPa and the temperature difference was 10.66 K, which was slightly larger than those of the specimen laminated +45°/−45° in directions. However, the temperature variation was greater than the temperature difference due to the impact test. These results were obtained due to the prior breakage of the specimen. In addition, the thermal image confirmed that the cracks propagated in the orthogonal direction from the impact site for the composite with 0°/90° configuration.

## 4. Conclusions

Unidirectional carbon fiber prepregs were laminated at three orientation angles, i.e., 0°, 0°/90°, and +45°/−45°, and the drop-weight impact test was conducted. After impact, the internal damage of the specimens was examined using the lock-in method, which is a non-destructive inspection method. Thereafter, the residual compressive strength was determined from the CAI test results, and the damage modes were confirmed using the IR thermal image camera. The conclusions of this study can be summarized as follows.

The results of the drop-weight impact test on the carbon composites at three different orientation angles revealed that the specimen laminated at angle of 0° absorbed the lowest impact energy, which resulted in the complete breakage of the fibers in the transverse direction. Although the epoxy resin supported the specimen laminated at an angle of 0° in the transverse direction, the damage occurred due to the susceptibility of the resin to impacts.

The drop-weight impact test results of the specimens oriented at angles of 0°/90° and +45°/−45° exhibited that more than 90% of the impact energy applied by the impactor were absorbed by the specimens, and these configurations were found to be effective in blocking the impact load of the impactor. The results can be attributed to the lamination of the fibers in orthogonal directions for both specimens.

The specimens after impact were analyzed using the lock-in method. The specimens were analyzed except the specimens laminated at an angle of 0° which underwent complete breakage during the impact test. The analyzed results revealed that there was internal delamination and cut fibers, in addition to the visible damage. In the residual compression test results, the temperature differences were not significant, given that the already-impacted specimens were subjected to the compression test. The specimen laminated at angles of 0°/90° exhibited higher residual compressive strengths than that the composite laminated at +45°/−45°.

## Figures and Tables

**Table 1:** Physical properties of UIN200C material.

Thickness (mm)	Fiber Areal Wt. (g/m^2^)	Fiber Content (vol.%)	Total Wt. (g/m^2^)
0.18	200	61.61	448

**Table 2:** Impact test results for specimens laminated in 0° direction.

Specimen No.	Max. Load (kN)	Total Impact Energy (J)	Absorbed Energy (J)	Impact Absorption Efficiency (%)	Maximum Temperature Difference (K)
1	5.00	37.74	4.15	10.99%	34.92
2	4.94	37.37	8.85	23.68%	38.35
3	4.69	37.19	8.28	22.26%	54.88
4	4.82	37.56	5.62	14.96%	55.94
Average	4.86	37.47	6.73	17.96%	46.02

**Table 3:** Impact test results for specimens laminated in +45°/−45° direction.

Specimen No.	Max. Load (kN)	Total Impact Energy (J)	Absorbed Energy (J)	Impact Absorption Efficiency (%)	Maximum Temperature Difference (K)
1	10.92	37.72	35.85	95.04%	3.96
2	10.52	37.33	34.53	92.50%	3.61
3	10.37	37.25	34.19	91.79%	3.36
4	10.39	37.78	33.66	89.09%	3.58
Average	10.55	37.52	34.56	92.11%	3.63

**Table 4:** Impact test results for specimens laminated in 0°/90° direction.

Specimen No.	Max. Load (kN)	Total Impact Energy (J)	Absorbed Energy (J)	Impact Absorption Efficiency (%)	Maximum Temperature Difference (K)
1	12.26	37.80	37.35	98.81	3.27
2	12.35	37.47	36.54	97.52	2.98
3	12.25	37.54	37.26	99.25	4.50
4	12.09	37.42	37.16	99.31	4.30
Average	12.24	37.56	37.08	98.72	4.01

**Table 5:** CAI test results of specimen according to the lamination angle.

Direction of Lamination	Specimens No.	Area (mm^2^)	Maximum Load (kN)	Residual Compressive Strength (MPa)	Temperature Difference (K)
+45°/−45°	1	540.73	37.56	69.46	4.48
	2	542.27	37.18	68.56	3.69
	3	540.73	36.58	67.65	3.41
	4	544.30	36.48	67.02	2.56
	Average	542.01	36.95	68.17	3.53
0°/90°	1	532.72	56.82	106.66	11.90
	2	538.46	54.32	100.88	8.94
	3	542.16	55.70	102.74	10.34
	4	537.35	61.14	113.78	10.66
	Average	537.67	57.00	106.01	10.46

## Data Availability

The data presented in this study are available on request from the corresponding author.
